# Supplementary material for: Investigation of the furcation morphology of permanent mandibular first molars by using micro-computed tomography
Source: BMC Oral Health. 2024 Sep 28;24:1150. doi: 10.1186/s12903-024-04892-5 (PMC11439296; doi:10.1186/s12903-024-04892-5)
Supplement: Supplementary file 1 — Supplementary Material 1 [file 12903_2024_4892_MOESM1_ESM.docx]

**Investigation of the furcation morphology of permanent mandibular first molars by using micro-computed tomography**

Yinfeng Qiu^1,2†^, Ying Tang ^3†^, Panpan Zhang ^2^, Jin Li ^1*^, Yongchun Gu^2^

1. Department of VIP Clinic, Nanjing Medical University, Affiliated Stomatological Hospital of Nanjing Medical University; Jiangsu Province Key Laboratory of Oral Diseases; Jiangsu Province Engineering Research Center of Stomatological Translational Medicine, Nanjing, China

2. Department of Dentistry, Suzhou Ninth People’s Hospital, Suzhou, China

3. Department of Pathology, Suzhou Ninth People’s Hospital, Suzhou, China

† Yinfeng Qiu and Ying Tang contributed equally to this work as first authors.

**Correspondence：**

Jin Li, Department of VIP Clinic, Nanjing Medical University, Affiliated Stomatological Hospital of Nanjing Medical University; Jiangsu Province Key Laboratory of Oral Diseases; Jiangsu Province Engineering Research Center of Stomatological Translational Medicine, Nanjing, China, 136 Hanzhong Road, Nanjing 210029, China.

Email: lijin6806@163.com

**Investigation of the furcation morphology of permanent mandibular first molars by using micro-computed tomography**

**Abstract**

**Background** To investigate the anatomic features of the root furcation of permanent mandibular first molars.

**Methods** A total of 50 extracted mandibular first molars (25 two-rooted and 25 three-rooted) were collected and scanned using micro-computed tomography. The digital models of teeth and root canal systems were reconstructed three-dimensionally. The tooth models were displayed in parallel projection mode from buccal and distal views. Screenshots were captured and subsequently analyzed using Image-Pro Plus 6.0 software after calibration. The furcation angle, root trunk length, maximum depth and level of distal root concaves of mesial roots, and length of enamel projections were measured, and the furcation types (classified into type V, type U and type W) were detected. Statistical analysis was performed using the Shapiro-Wilk's test, one-way analysis of variance, Student’s *t-*test and Chi-square test.

**Results** The mean furcation angle between the distobuccal (DB) and distolingual (DL) roots (in distal view) was the greatest (59.2°), whereas the furcation angle between the mesial and DL roots (in buccal view) was the smallest (25.4°) among the four furcation angles (all *p* < 0.05). Regarding the furcation types, bucco-lingual root trunk length, maximum depth and site of the distal root concavities, and enamel projection length, no significant differences were detected between the three- and two-rooted molar groups (all *p* > 0.05). The frequency of type V was the highest (54.0%), followed by type U (26.0%), and type W had the lowest occurrence rate (20.0%). The mean length of distal root trunk in the three-rooted mandibular molars was significantly greater than that of the buccal/lingual one (3.7 mm vs. 3.0 mm, *p* < 0.01). The maximum depth of the distal concavities of the mesial roots was on average 0.66 ± 0.19 mm, and the site was located at an average of 2.8 ±1.3 mm below furcation. The mean length of buccal enamel projections was significantly longer than that of lingual ones (3.1 mm vs. 0.7 mm, *p* < 0.01).

**Conclusions** The furcation anatomy of the mandibular first molar is complex, and the presence of the DL root may further complicate its topography. A thorough understanding of these anatomic features is essential for successful periodontal treatment.

**Key words** X-ray microtomography, Tooth root, Molar, Furcation defects, Dental enamel

**Background**

Periodontal disease, characterized by the destruction of alveolar bone and loss of connective tissue attachment mainly by oral bacterial infection, is the primary cause of tooth loss in adults [1]. The purpose of therapeutic procedures, whether surgical or non-surgical, is the elimination of supra- or subgingival bacterial deposits from the root surface and the prevention of their recurrence, and effective mechanical debridement of root surface is critically important for active and maintenance periodontal therapy [2]. However, the treatment of periodontally diseased molars with furcation involvement (FI) is a challenging task in the field of clinical periodontology, and it has been reported that molars with FI are at a higher risk of tooth loss [2-6]. The root furcation morphology is one of the critical factors that may affect the diagnosis, treatment and prognosis of FI [7,8]. Previous scholars demonstrated that the furcation anatomy of mandibular first molars is complex, with attentions mainly focused on the furcation divergence, furcation entrance size, root trunk length, root concavities, furcation ridges, enamel projections, etc., which may affect effective plaque control by both patients and professionals [7,9-12]. A small angulation of root divergence and a long root trunk typically indicate that the furcation entrance is narrow and located more apically, which hinders the accessibility of the instrument through the furcation entrance. The presence of root concaves at the furcation side of the mesial or distal roots, as well as the furcation ridges, creates an environment favorable to bacterial plaque retention, and the effectiveness of instrumenting the furcation area may be compromised because conventional curettes do not easily fit into these areas [13]. Moreover, the mesial root of mandibular molars is inherently curved towards the distal side [14], and the canals are not located centrally in the root, but lie closer to the furcation side and distal root concavity [15,16]. Abou-Rass et al. [16] first termed the thinner distal dentin wall the “danger zone”, as this area is prone to strip perforation during root canal instrumentation. Cervical enamel projection (CEP) is defined as a dipping of enamel from the cementum-enamel junction of molars towards, and often into the furcation area, and a large number of studies demonstrated that this developmental anomaly is an etiologic factor in FIs [17]. Therefore, information about the prevalence, location and extension of CEPs is crucial for clinicians to draw treatment plan or make the prognosis.

The mandibular first molar normally has two roots, but in some cases, a third root can be detected at the distolingual (DL) side of the teeth. This root variation is an important ethnic trait for Mongolian populations, with a high prevalence rate ranging from 5% to 40%; while in the black and white populations, the frequency is often below 5% [14,15,18]. A recent multinational study revealed that the global occurrence of the third root is approximately 3% [19]. The DL root is usually conical and severely curved, and may pose difficulty on root canal preparation, and it has been extensively studied in the field of endodontology [14]. From a periodontological perspective, Huang et al. [20] reported that in a selected population in Taiwan, significantly greater probing depths and attachment loss were detected in the DL sites of 3RM1s compared to 2RM1s in the category of advanced periodontitis, indicating that the presence of a DL root may contribute to localized periodontal destruction. However, the anatomic features of the root furcation associated with of this root variation have not been fully investigated.

Periapical and panoramic radiographs are conventional radiographic methods frequently used to estimate the furcation lesions and root morphology. However, these methods have inherent limitations as they provide only two-dimensional images, and may cause overlapping structures and image distortion. Cone beam computed tomography (CBCT) is three-dimensional and is currently a more reliable imaging technique (gold standard) for clinical diagnosis of FIs due to its high resolution and non-destructive nature [1,21]. Micro-computed tomography (micro-CT) provides even higher resolution, approximately 10 times greater than CBCT, and has been widely used in ex vivo studies of human teeth [14]. Supported by built-in programs or third-party software, digital three-dimensional (3D) tooth models can be reconstructed, which allows for both quantitative and qualitative analysis of the complicated furcation morphology. The purpose of this study is to investigate the anatomic characteristics of the root furcation in mandibular first molars using micro-CT.

**Materials and Methods**

**Collection of sample teeth**

Ethics approval of this study was granted by the Ethics Committee of Suzhou Ninth People’s Hospital (Issuing Number: KY2022-089-01). All subjects were native Chinese, and the teeth were extracted because of periodontal disease, non-restorable caries, trauma, or prosthodontic reasons. The tooth type (the permanent mandibular first molars) was accurately identified by the operator soon after tooth extraction, and the age of the subject was also recorded. The exclusion criteria were as follows: (a) teeth with open root apices, (b) teeth that had been previously treated endodontically (as the root canal filling materials may cause imaging artifacts, potentially affecting the accuracy of odontometric measurements), (c) teeth with root caries, fractures, internal or external resorption. G*Power software (ver. 3.1.9.7; Heinrich-Heine-Universität Düsseldorf, Düsseldorf, German) was used to calculate the sample size. According to the analysis, a minimum of 26 samples per tooth group (two- or three-rooted molars) was required to achieve an effect size of 0.80 in 80% power and 95% confidence intervals. Since the DL root is prone to fracture during tooth extraction, collection of a large sample size of ex vivo three-rooted MFMs without major defection is a challenging task. Utimately, a total of 50 permanent mandibular first molars (25 were two-rooted [2RM1] and 25 were three-rooted [3RM1]) were included in the current study. The ages of the subjects ranged from 18 to 79 years (mean age = 48.8 ± 16.2 years).

**Micro-CT scanning**

The sample teeth were scanned using a micro-CT device (SkyScan1174; Bruker-microCT, Kontich, Belgium). The parameters used were: voxel size of 18.7 μm, energy of 800 mA and 50 kVp, 180˚ of rotation with 0.7˚ increments, and 1-mm-thick aluminum filter. Then, the micro-CT scan data were uploaded to Mimics 21.0 software (Materialise, Leuven, Belgium) for 3D reconstruction of the teeth and root canal systems.

**Odontometric analysis**
In the Mimics interface, the 3D tooth models were displayed in a parallel projection mode [15]. In this mode, the principle of imaging was the same as that of radiographic approach (parallel cone technique), which minimizes distortion and allows for accurate odontometric measurements. The tooth models were displayed in buccal view and distal view, respectively. In the buccal view, the position of the tooth model was further adjusted (by rotation) until the images of the mesiobuccal (MB) and mesiolingual (ML) canals overlapped (in the buccal view of the mesial root) (Fig.1A). Then, screenshots in both views were saved in TIFF format and analyzed using Image-Pro Plus 6.0 software (Media Cybernetics, Silver Spring, MD, USA). After calibration, the following parameters were measured:

1. Angle of the root furcation: As shown in Fig. 1, the angles formed between the mesial and distal/DB roots (α/α1), and mesial and DL roots (β) were measured in the buccal view. The furcation angle between the DB and DL roots (γ) was measured in the distal view.
2. Furcation type and root trunk length: In the distal view, the transparency of the tooth models were set to be semi-transparent, and the contours of furcation roofs could completely be displayed, and were classified into three types: in type V, the intermediate ridge projected and formed a convex in the inter-radicular area (Fig. 2A). In type U, the furcation roof in the inter-radicular area was flat, and neither protrusion nor depression was detected (Fig. 2B). In type W, a concavity was formed at the center of inter-radicular area, demarcated by buccal and lingual furcation ridges (Fig. 2C). The vertical distance from the lowest point (vertex) of furcation ridge to the cement-enamel junction (CEJ) plane was measured as the bucco-lingual root trunk length, and the vertical distance from the fornix of distal furcation to the CEJ plane was the length of distal root trunk.
3. Depth and location of the distal root concavity of the mesial root: In the buccal view, the position of the tooth model (in semi-transparent mode) was further adjusted until the depth of distal concavity of the mesial root could be maximally displayed. The screenshots were saved to determine the site and value of the maximum depth of the distal concavity. Its distance to furcation (L_1_), as well as to the apex (L_2_), was measured along the canal, and L_1_/(L_1_+L_2_) × 100% was calculated to describe the level of maximum depth (Fig. 3).
4. Length of buccal and lingual enamel projections: In Mimics software, on the 3D tooth model, a spline curve was drawn on the tooth surface from the CEJ to the tip of the buccal/lingual enamel projection (Fig. 4). The length of the spline curve was taken as the length of enamel projection. The presence of enamel pearls was also recorded.

All morphologic assessment and measurements were performed by one examiner (*Y. Q*). An expert endodontist (*Y. G*) and the observer (*Y. Q*) performed the calibration. In the pilot study, the observer was trained and calibrated to identify the type of root furcation using micro-CT images of 20 teeth (10 2RM1s and 10 3RM1s). Disagreements were discussed, until a consensus was reached. Cohen's kappa test was used to evaluate the inter- and intra-observer errors. Each examiner evaluated the same 20 teeth twice independently, at an interval of 14 days. The intra-observer kappa value was 1.0 for one observer (*Y. G*) and 0.9 for another (*Y. Q*), and the inter-observer kappa value was 0.9 (all *p* = 0.000), suggesting the inter- and intra-observer agreement were both excellent.

**Statistical analysis**

SPSS 17.0 software (SPSS, Chicago, IL, USA) was used for all statistical analyses. The normality of the data was evaluated with the Shapiro-Wilk test. After verification of data distribution, the one-way analysis of variance (ANOVA) and the post hoc LSD test were used for multiple group comparisons, and Student’s *t-*test was used for comparisons between two groups. Chi-square test was used to compare the frequencies. The significance level was established at 5%.

**Results**

The measurement results of the furcation angles in mandibular first molars are summarized in Table 1 and Fig. 5. The mean furcation angle between the DB and DL roots was the greatest (59.2°) as compared with the other three furcation angles (*p* all < 0.01), while the separating angle between the mesial and DL roots was the smallest (25.4°), and was significantly (*p* < 0.05) less than that between the mesial and DB roots. In five (20%) 3RM1s, the DL root was completely overlapped by the DB root in the buccal view, and in no case, the DL root was exposed (even partially) at the distal side of DB root.

The occurrences of the three types of furcation are shown in Tables 2, S1 and S2. The difference between the two- and three-rooted groups had no statistical significance (*X*^2^ = 0.114, *p* = 0.945). Among all the sample teeth, type V furcation accounted for the largest proportion of 54.0%, followed by type U (26.0%), and type W furcation accounted for the smallest proportion of 20.0%. The Chi-square test revealed no statistically significant differences between genders (Table S1) or between the two age groups (<50 years vs. ≥50 years) (Table S2). The measurement results of root trunk length were listed in Table 3. The mean length of the distal root trunk was significantly longer than the buccal/lingual one (3.7 mm vs. 3.0 mm, *p* < 0.01).

Table 4 lists the measurement results for the maximum depth and location of the distal concavities of the mesial roots. There was no significant difference between the two- and three-rooted tooth groups (*p* > 0.05). Among the total sample teeth (*n* = 50), the maximum depth of the distal root concavities was (0.66 ± 0.19) mm, and site was located at an average of (2.8 ±1.3) mm below the furcation or coronal (31.4 ±14.2) % of the mesial root length.

The measurement results of the enamel projections are shown in Table 5 and Fig. 6. The mean length of the enamel projections at the buccal side were significantly longer than that at the lingual side (3.1 mm vs. 0.7 mm, *p* < 0.01). No enamel projection was detected at the distal side. Enamel pearls were detected in two 2RM1s and two 3RM1s, with a total occurrence rate of 8.0% (4/50).

**Discussion**

FI shows the greatest occurrence rate on mandibular first molars, and its main clinical manifestations include the formation of periodontal pocket, loss of connective tissue attachment and intra-radicular bone [22]. Previous studies demonstrated that the complexity of furcation anatomy is an important contributory factor in the progress of furcation lesions [3,7].

In this study, we found that the separating angle between the DB and DL roots of 3RM1s (in the distal view) is the greatest, and the mean value (~60°) is approximately two-folds larger than that between mesial and distal/DB roots in the buccal view. According to an in vivo CBCT study on a Hong Kong population, Ho et al. [23] found the vertical separation angle between DB and DL roots was 62.8° ± 11.4°, which is very close our data. Another CBCT investigation on Chinese children indicated that the DL root could also occur in deciduous mandibular second molars, and the distal furcation angle was 67.4° ± 14.4°, significantly greater than that of the permanent 3RM1s [24]. The great separating angle between DB and DL roots indicates that the orientation of the endodontic instrument may significantly deviate from the axis of the tooth as it is inserted into the DB/DL canal, and overzealous drilling or instrumentation may easily lead to perforation at the pulp floor or root furcation area. Additionally, care should be taken to preserve pericervical dentin during coronal preflaring or post space preparation,

as a wider root separation increases the vulnerability to root fractures. Table 1 shows that the angle between the mesial and DL roots is the smallest, and is even significantly less than the angle between mesial and DB roots. This finding also suggests that the DL root is generally located at the mesial side of the DB root in the mesio-distal direction. A deep understanding of the location and morphology of DL roots can be useful for clinicians to carry out furcation probing or periodontal treatment on 3RM1s with FI. In the buccal view, although only one fifth of the DL root was completely overlapped by the DB root, it is still prone to missed diagnosis or misdiagnosis on conventional radiographs due to superimposition of adjacent alveolar bone, and the use of multiple angled radiographs or CBCT [1,23] allows for more reliable detection of this root trait. Wang et al. [25] carried out an ex vivo experiment to evaluate the X-ray projection angulation for successful detection of the extra DL root in 3RM1s (*n* = 25). On the orthoradial radiographs, correct assessment could only be found in 7 teeth (28%), and in the other 18 specimens, the DL roots were moderately (*n* = 8) or severely (*n* = 10) overlapped by the DB roots; while an additional 25° mesial horizontal angulation radiograph, but not the 25° distal angulation radiograph, yielded a 100% correct assessment rate [25]. Recently, convolutional neural network based deep learning system was applied for the detection of 3RM1s on panoramic radiographs, significantly improving diagnostic accuracy compared to that of clinicians [26]. The presence of the DL root could affect the efficiency of the periodontal instrumentation and plaque control, and a small separation angle was unfavorable to gaining access for debridement in the furcation area [23,27].

To examine the furcation ridges, Everett et al [27] divided 328 extracted mandibular molars into three groups, and by grinding off the mesial/distal half of the tooth, or amputating the tooth roots, the root furcations were observed under the microscope in three different views. For the first time they reported the presence of a distinct “intermediated bifurcational ridge”, as well as the “buccal and lingual bifurcation ridges”. Our current study was non-invasive, and by modifying the transparency of the tooth model into semi-transparent, the anatomic features of the furcation roof could be displayed vividly (Fig. 2). In the proximal (distal) view, the type V furcation accounted for nearly half of the total teeth, and a pronounced intermediate ridge could be detected; while in the study of Everett et al. [28], intermediated bifurcational ridge was found to be pronounced in 44%, noticeable in 29%, and absent in 27%, and primarily consisted of cement. In regard to Type W furcation, which only accounted for one fifth of our sample teeth, the buccal and lingual ridges were located near the buccal and lingual entrance of the furcation, respectively, which were mainly formed by dentine and covered with a thin layer of cement [28]. In regard to type U furcation (approximately accounting for one fourth of total teeth), the buccal and lingual ridges were not apparent, and neither protrusion nor furcation concave was formed. Everett et al. [28] reported that in 37% of cases, there was no noticeable differences between the buccal and lingual ridges or these ridges were not apparent. The concave at the center of inter-radicular area may act as an ecological niche for biofilm, and would pose challenge for proper debridement [28,29]. In the current study, neither gender nor age was found to significantly influence the proportion of furcation types (Table S1, and S2), although both factors may affect cement deposition and furcation configurations. Further studies with larger sample sizes and a more diverse age range are warranted. The anatomical complexity of furcation ridges presents significant challenges in diagnosing periodontal involvement. Clinicians must consider these structures carefully when assessing FI using clinical and radiographic methods. Precise identification of the furcation type is crucial for effective treatment planning, which may include procedures such as scaling and root planing, furcation debridement, or surgical interventions.

In terms of root trunk length, earlier studies reported that molars with short root trunks were susceptible to FI due to the increased likelihood of plaque retention, and conversely, a longer root trunk meant that the furcation area was located deeper within the bone, which may offer some protection against early FI in periodontal disease. [9,10]. In restorative dentistry, root trunk length can influence the design and success of restorations. Particular attention is required when restoring teeth with shorter root trunks to avoid exacerbating periodontal issues. However, FI detected in molars with long root trunks means an advanced stage of periodontitis, difficulties in diagnosis and treatment, and a poor prognosis [7]. The current study found that the mean distal trunk length is 0.7 mm longer than that of the buccal/lingual root trunk (3.0 mm). Our data is lower than those (distal trunk: 5.2 mm; buccal/lingual trunk: 4.0 mm) reported by Ho et al. [23], and the discrepancy can be due to the differences in the research method and racial populations. A short root trunk is mandatory when the clinician considers root resection or tunneling procedures as treatment options for molars with FI, while molars with long root trunks are unsuitable for such procedures [30]. Hou et al. [10] proposed a classification of molar FI based on root trunk and horizontal and vertical attachment loss, and the root trunks were classified into 3 types according to the ratio of vertical length of root trunk to root length (types A, B and C indicate root trunk involving the cervical third, the cervical half, and the cervical two thirds of roots, respectively), which were associated with guidelines in diagnosis and treatment of FI. When performing periodontal surgery, such as flap surgery or regenerative procedures, the length of the root trunk must be considered. Shorter root trunks may require more precise surgical techniques to effectively manage furcation involvement and to ensure adequate healing and bone regeneration.

Root concavities are clinically significant because they increase the root’s surface area, thereby aiding in the tooth's stability within the alveolar bone. However, previous scholars have also demonstrated that root concavities may serve as an ecological niche for bacterial plaque and contribute to the formation of deeper periodontal pockets [30]. After surgical periodontal treatment, plaque, calculus and contaminated cementum should be removed adequately by periodontal instruments, and a smooth root surface that is more biologically acceptable to soft tissue should be created, which can ensure long-term fate of the involved teeth [27]. The presence of the root concavity may compromise the treatment outcome due to its inaccessibility to cleaning. Additionally, deep root concavities may predispose the tooth to fractures, particularly if significant bone loss occurs around the tooth. This is especially relevant in molars subjected to high occlusal forces. Root concavities often cannot be detected in the conventional buccolingual radiographs [31,32], while in vivo CBCT examination [33] or ex vivo micro-CT imaging can three-dimensionally visualize its detailed configuration. To assess the depth of root concaves, many previous studies, based on tooth sections or micro-CT/CBCT scans, frequently took measurements in several or a series of horizontal (axial) root slices [16,33,34], and therefore, the measurement data were discrete along the root. In the current study, in buccal view of the mesial root, the continuous distribution of the distal concavity depth, as well as the corresponding canal wall thickness in the mesio-distal direction, could be displayed along the root length in one screenshot (Fig. 3). We found there was no difference between the three- and two-rooted tooth groups; averagely, the maximum depth was located at 2.8 mm below furcation or the coronal third level of the whole root length. While several other scholars arbitrarily defined that in the mesial roots of mandibular molars, the distal furcal root dentine 2 mm below furcation was the danger zone [35,36]. Based on CBCT images, Bolbolian et al. [37] measured the dentin thickness and depth of distal concavity of the mesial roots from the furcation to 5 mm below. The area with the greatest depth of concavity was used to calculate the minimum dentin thickness and regarded as the danger zone. They demonstrated that danger zone was in the range of 0 to 3 mm below furcation with a probability of 93.4% [37]. However, the current data indicate that the maximum depth of the distal concavity does not always correspond to the minimum canal wall thickness along the root (Fig. 3B). In determining the site of danger zone, the canal curvature, dentine wall thickness, and distal root concavity should all be considered, and this issue deserves further investigations.

Ectopic enamel can be detected on the root either as cervical enamel projection, or by enamel pearl, which can induce accumulation of plaque, and are associated with rapid progression of pocket formation, periodontal attachment loss and occurrence of FI [38]. The prevalence of enamel projections can be influenced by ethnicity; it ranges from 8.6% to 85% worldwide [39], and Asian subjects have a higher prevalence rate as compared with other races [30]. Hou et al. [40] reported that the mandibular first molar exhibited the highest occurrence rate among different molars, while Grewe et al. [41] reported the most common site was the buccal side of mandibular second molars. Similar to coronal enamel, the fibers of the periodontal ligament are unable to attach to enamel projections [42]. When guided tissue regeneration is performed on molars with FI, the enamel projections should be removed via enameloplasty for a better outcome [11]. Masters and Hoskinsdean [43] classified the severity of these projections into three grades: Grade 1, short but distinct change in contour of CEJ toward furcation; Grade 2, the enamel projection approaches furcation, but no actually making contact with it; Grade 3, the enamel projection extends into the furcation; while the current study allows for accurate quantitative assessment of the 3D length of enamel projections. Table 5 shows that the mean length of the buccal enamel projections is significantly longer than that of the lingual ones, suggesting that our concerns should be put on the buccal side. Its length varies over a wide range from 0.65 to 6.47 mm, which indicates that the dentist should evaluate the status of cervical enamel projections (CEPs) for each patient carefully, and individualized clinical management of this dental anomaly should be considered regarding the eminent individual difference in severity of CEPs. The treatment decisions also depend the specific situation of the subject [17]. Fig. 6 shows more than half of the buccal enamel projects are longer than 3 mm; considering the mean buccal/lingual root trunk length is also 3 mm, we estimate that Masters and Hoskinsdean’s Grade 3 may account for the largest proportion. These data can be useful for clinicians in management of CEPs during periodontal treatment, especially for treatments of Chinese patients. It is difficult to accurately determine the severity of enamel projections clinically, especially for those periodontally-healthy molars. Theoretically, CBCT imaging can best display the extent of enamel projections in the furcation, though CBCT is not routinely performed to assess furcation due to the extra radiation dose exposed and its use should apply with the principle of ALARA (as low as reasonably achievable).

Previous studies have demonstrated that both gender and age can influence the root morphology. However, concerning the prevalence of 3RM1s, gender differences have generally not been observed [44], a finding corroborated by our recent in vivo study based on CBCT examinations [24]. As shown in Tables S1 and S2, neither gender no age didn’t significantly impact the incidence of furcation types, which may be attributed to the small sample size. Age, along with various pathological factors, may impact the deposition of cementum in the furcation area, thereby altering furcation morphology (e.g., the thickness of furcation ridges and the depth of root concaves). In this study, the mean age of subjects was 48 years, with a significant proportion of the sample teeth collected from elderly patients. Thus, our results are particularly relevant for guiding dental treatments in elderly patients.

This study has several limitations. First, all the sample teeth were collected from a Chinese population. Since ethnicity is an important influencing factor, further studies on teeth from other ethnic populations are essential to validate our conclusions. Second, the DL roots are often curved and tiny, and prone to fracture during tooth extraction, which may result in small sample size and introduce bias into the conclusions. In the future, in vivo CBCT studies with larger sample sizes may mitigate this limitation, and the impacts of the age or pathological factors can also be analyzed. Third, although all odontometric analyses were performed on 3D tooth models reconstructed from high-resolution micro-CT scans, to simplify the measurement process, the furcation angle, canal curvature, and furcation type were evaluated in the buccal-lingual view and/or proximal view, which does not constitute a true “3D” measurement. Finally, micro-CT images are unable to clearly distinguish between cementum and dentine in the furcation areas, while pathological factors and the aging processes may affect the deposition or absorption of the cement, thereby altering root furcation morphology. Clinicians should be aware of these limitations when comparing our findings with other studies or applying them to clinical practice.

**Conclusions**

Within the limitations of this study, it can be concluded that the furcation anatomy of the mandibular first molar is complex, and the presence of a DL root may further complicate its topography. A thorough understanding of these anatomic features is essential for successful periodontal treatment.

**Abbreviations**

3D three-dimensional

ALARA as low as reasonably achievable

CBCT cone-beam computed tomography

CEJ cement-enamel junction

CEP cervical enamel projections

DB disto-buccal

DL disto-lingual

FI furcation involvement

GTR guided tissue regeneration

MB mesiobuccal

ML mesiolingual

micro-CT micro-computed tomography

**Declarations**

**Ethics approval and consent to participate**

The human mandibular first molars used in this study were obtained from the Department of Dentistry, Ninth People’s Hospital of Suzhou. All methods have been performed in accordance with the Declaration of Helsinki and have been approved by the Ethics Committee of Ninth People’s Hospital of Suzhou with the approval number # KY2022-089-01. Informed consent was obtained from all subjects and/or their legal guardian(s).

**Consent for publication**

Not applicable.

**Availability of data and materials**

All the datasets used and analyzed during the current study are available from the corresponding author on reasonable request.

**Competing interests**

The authors declare that they have no competing interests.

**Funding**

This study was funded by Suzhou “Medical Health Science and Technology Innovation” Project (SKY2022030).

**Authors’ contributions**

J.L. and Y.G. contributed to the study’s conception and design. Data collection was performed by Y.Q., Y.T., and P.Z.; Y.Q. and Y.T. contributed to the analysis or interpretation of data. Statistical analysis was performed by Y.T. and P.Z. The main manuscript text was written by Y.Q. and Y.T. All authors reviewed the manuscript.

**Acknowledgments**

Not applicable

**Authors details**

^1^ Department of VIP Clinic, Nanjing Medical University, Affiliated Stomatological Hospital of Nanjing Medical University; Jiangsu Province Key Laboratory of Oral Diseases; Jiangsu Province Engineering Research Center of Stomatological Translational Medicine, Hanzhong Road 136#, 210039 Nanjing, China.^2^ Department of Dentistry, Ninth People’s Hospital of Suzhou, Soochow University, Ludang Road 2666#, Wujiang Dist., 215200 Suzhou, China.^3^ Department of Pathology, Ninth People’s Hospital of Suzhou, Soochow University, Ludang Road 2666#, Wujiang Dist., 215200 Suzhou, China.

**References**

1. Gonçalves BC, Costa ALF, Correa R, Andere NMRB, Ogawa CM, Santamaria MP, et al. Analysis of geometrical tomographic parameters of furcation lesions in periodontitis patients. Heliyon. 2021;7(1): e06119.
2. Roussa E. Anatomic characteristics of the furcation and root surfaces of molar teeth and their significance in the clinical management of marginal periodontitis. Clin Anat, 1998,11(3):177-86.

3. Hamp SE, Nyman S, Lindhe J. Periodontal treatment of multi rooted teeth. Results after 5 years. J Clin Periodontol,1975, 2(3):126-135.

4. Trullenque-Eriksson A, Tomasi C, Petzold M, Berglundh T, Derks J. Furcation involvement and tooth loss: A registry-based retrospective cohort study. J Clin Periodontol,2023,50(3):339-347.

5. Pretzl B, Kaltschmitt J, Kim TS, Reitmeir P, Eickholz P. Tooth loss after active periodontal therapy. 2: tooth-related factors. J Clin Periodontol,2008,35(2):175-182.

6. Nibali L, Sun C, Akcalı A, Yeh YC, Tu YK, Donos N. The effect of horizontal and vertical furcation involvement on molar survival: A retrospective study. J Clin Periodontol,2018,45(3):373-381.

7. Hou GL, Hung CC, Tsai CC, Weisgold AS. Topographic study of root trunk type on Chinese molars with Class III furcation involvements: molar type and furcation site. Int J Periodontics Restorative Dent,2005,25(2):173-179.

8. Limiroli E, Calò A, Limiroli A, Cortinovis I, Rasperini G. Radiographic ratios for classifying furcation anatomy: proposal of a new evaluation method and an intra-rater and inter-rater operator reliability study. Clin Oral Investig,2023, 27(4):1541-1546.

9. Hou GL, Tsai CC. Types and dimensions of root trunk correlating with diagnosis of molar furcation involvements. J Clin Periodontol,1997,24(2):129-135.

10. Hou GL, Cheng YM, Tsai CC, Weisgold AS. A new classification of molar furcation involvement based on the root trunk and horizontal and vertical bone loss. Int J Periodontics Restorative Dent,1998,18(3):257-265.

11. Ko MJ, Cho CM, Jeong SN. Characteristics of the molar surface after removal of cervical enamel projections: comparison of three different rotating instruments. *J* Periodontal Implan,2016, 46(2):107-115.

12. Pepelassi EM, Bissada NF, Greenwell H, Farah CF. Doxycyclinetricalcium phosphate composite graft facilitates osseous healing in advanced periodontal furcation defects. J Periodontol. 1991;62(2):106-15.

13. Marcaccini AM, Pavanelo A, Nogueira AV, Souza JA, Porciúncula HF, Cirelli JA. Morphometric study of the root anatomy in furcation area of mandibular first molars. J Appl Oral Sci. 2012;20(1):76-81.

14. Gu Y, Lu Q, Wang P, Ni L. Root canal morphology of permanent three-rooted mandibular first molars: Part II--measurement of root canal curvatures. J Endod,2010,36(8):1341-1346.

15. Wu MK, van der Sluis LW, Wesselink PR. The risk of furcal perforation in mandibular molars using Gates-Glidden drills with anticurvature pressure. Oral Surg Oral Med Oral Pathol Oral Radiol Endod,2005,99(3):378-382.

16. Abou-Rass M, Frank AL, Glick DH. The anticurvature filling method to prepare the curved root canal. J Am Dent Assoc,1980,101(5):792-794.

17. Kim HJ, Kim SY. Cervical enamel projections from a periodontal perspective: A scoping review. Clin Anat. 2024;37(3):353-65.

18. Qiao X, Zhu H, Yan Y, et al. Prevalence of middle mesial canal and radix entomolaris of mandibular first permanent molars in a western Chinese population: an in vivo cone-beam computed tomographic study. BMC Oral Health, 2020, 20(1):224.

19. Hatipoğlu FP, Mağat G, Hatipoğlu Ö, et al. Assessment of the prevalence of radix entomolaris and distolingual canal in mandibular first molars in 15 countries: a multinational cross-sectional study with meta-analysis. J Endod, 2023,49(10):1308-1318.

20. Huang RY, Lin CD, Lee MS, et al. Mandibular disto-lingual root: a consideration in periodontal therapy. J Periodontol,2007,78(8):1485-1490.

21. Qiao J, Wang S, Duan J, Zhang Y, Qiu Y, Sun C, et al. The accuracy of cone-beam computed tomography in assessing maxillary molar furcation involvement. J Clin Periodontol. 2014; 41(3):269-74.

22. Kinane DF, Stathopoulou PG, Papapanou PN. Periodontal diseases. Nat Rev Dis Primers.2017; 3:17038.

23. Ho DKL, Wong JHL, Pelekos G, Deng K, Tonetti MS. Prevalence and morphological characteristics of disto-lingual roots in mandibular first molars: a cone beam CT study with diagnostic and therapeutic implications. Clin Oral Investig.2021;25(6):4023-4030.

24. Jiang C, Pei F, Wu Y, Shen Y, Tang Y, Feng X, et al. Investigation of three-rooted deciduous mandibular second molars in a Chinese population using cone-beam computed tomography. BMC Oral Health. 2022;22(1):329.

25. Wang Q, Yu G, Zhou XD, Peters OA, Zheng QH, Huang DM. Evaluation of x-ray projection angulation for successful radix entomolaris diagnosis in mandibular first molars in vitro. J Endod.2011;37(8):1063-8.

26. Hiraiwa T, Ariji Y, Fukuda M, Kise Y, Nakata K, Katsumata A, et al. A deep-learning artificial intelligence system for assessment of root morphology of the mandibular first molar on panoramic radiography. Dentomaxillofac Radiol. 2019;48(3):20180218.

27. Bower RC. Furcation morphology relative to periodontal treatment. Furcation entrance architecture. J Periodontol.1979; 50(1): 23‑27.

28. Everett FG, Jump EB, Holder TD, Williams GC. The intermediate bifurcational ridge: a study of the morphology of the bifurcation of the lower first molar. J Dent Res,1958,37(1):162-169.

29. Santana RB, Uzel MI, Gusman H, Gunaydin Y, Jones JA, Leone CW. Morphometric analysis of the furcation anatomy of mandibular molars. J Periodontol,2004,75(6):824-829.

30. Goh EXJ, Ong MMA. Anatomical, microbiological, and genetic considerations in treatment of Chinese periodontal patients. J Investig Clin Dent,2019, 10(1):e12381.

31. Walter C, Kaner D, Berndt DC, Weiger R, Zitzmann NU. Three-dimensional imaging as a pre-operative tool in decision making for furcation surgery. J Clin Periodontol,2009,36(3):250-257.

32. Zhou G, Leng D, Li M, et al. Root dentine thickness of danger zone in mesial roots of mandibular first molars. BMC Oral Health, 2020,20(1):43.

33. Berutti E, Fedon G. Thickness of cementum/dentin in mesial roots of mandibular first molars. J Endod,1992,18(11):545-548.

34. De-Deus G, Rodrigues EA, Belladonna FG, et al. Anatomical danger zone reconsidered: a micro-CT study on dentine thickness in mandibular molars. Int Endod J,2019,52(10):1501-1507.

35. Zhou G, Leng D, Li M, Zhou Y, Zhang C, Sun C, et al. Root dentine thickness of danger zone in mesial roots of mandibular first molars. BMC Oral Health.2020; 20(1):43.

36. Sauáia TS, Gomes BP, Pinheiro ET, Zaia AA, Ferraz CC, Souza-Filho FJ, et al. Thickness of dentine in mesial roots of mandibular molars with different lengths. Int Endod J.2010; 43(7):555-9.

37. Bolbolian M, Ramezani M, Valadabadi M, Alizadeh A, Tofangchiha M, Ghonche MRA, et al. Dentin thickness of the danger zone in the mesial roots of the mandibular molars: A cone beam computed tomography analysis. Front Biosci (Schol Ed).2023;15(1):3.

38. Hou GL, Tsai CC. Cervical enamel projection and intermediate bifurcational ridge correlated with molar furcation involvements. J Periodontol,1997,68(7): 687-693.

39. Chan HL, Oh TJ, Bashutski J, Fu JH, Wang HL. Cervical enamel projections in unusual locations: A case report and mini-review. J Periodontol,2010,81(5):789-795.

40. Hou GL, Tsai CC. Relationship between periodontal furcation involvement and molar cervical enamel projections. J Periodontol,1987,58(10):715-721.

41. Grewe JM, Meskin LH, Miller T. Cervical enamel projections: Prevalence, location, and extent; With associated periodontal implications. J Periodontol, 1965,36(6):460-465.

42. Blanchard SB, Derderian GM, Averitt TR, John V, Newell DH. Cervical enamel projections and associated pouch-like opening in mandibular furcations. J Periodontol,2012,83(2):198-203.

43. Masters DH, Hoskins SW Jr. Projection of cervical enamel into molar furcations. J Periodontol,1964,35(1):49-53.

44. Turner, CG. Three-rooted mandibular first permanent molars and the question of American Indian origins. Am J Phys Anthropol, 1971,34 (2): 229-241.

**Figures:**

**
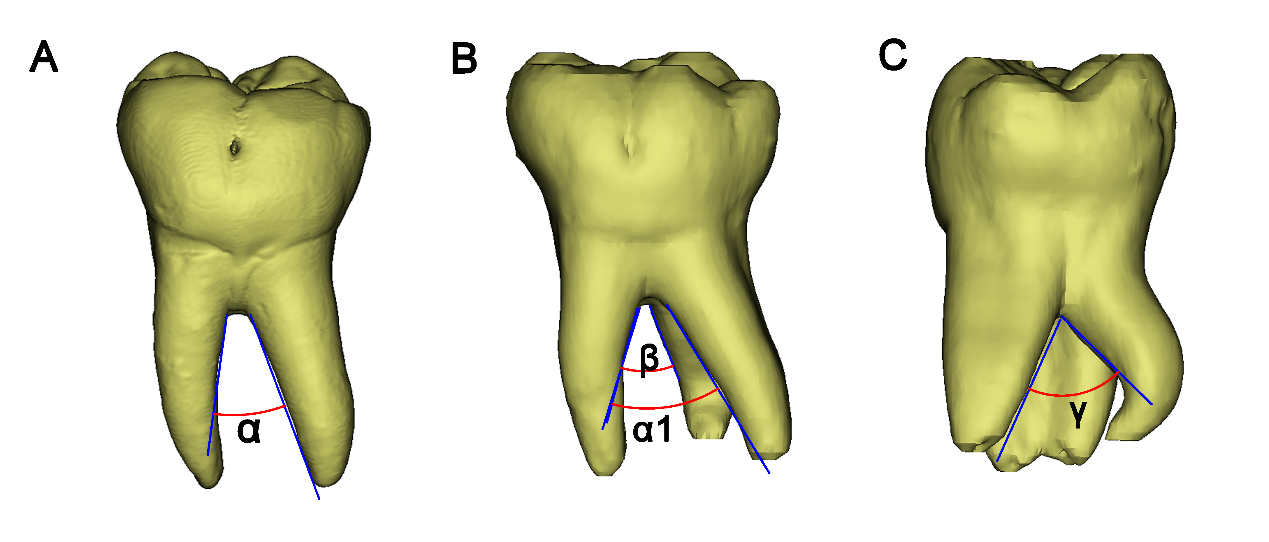
**

**Fig. 1** Measurement of the furcation angles in mandibular first molars. **a** Furcation angle between the mesial and distal roots of a two-rooted mandibular (α). **b** Furcation angles between the mesial and DB roots (α1), and mesial and DL roots (β) of a three-rooted mandibular molar. **c** Furcation angle between the DB and DL roots (γ) of a three-rooted mandibular first molar.


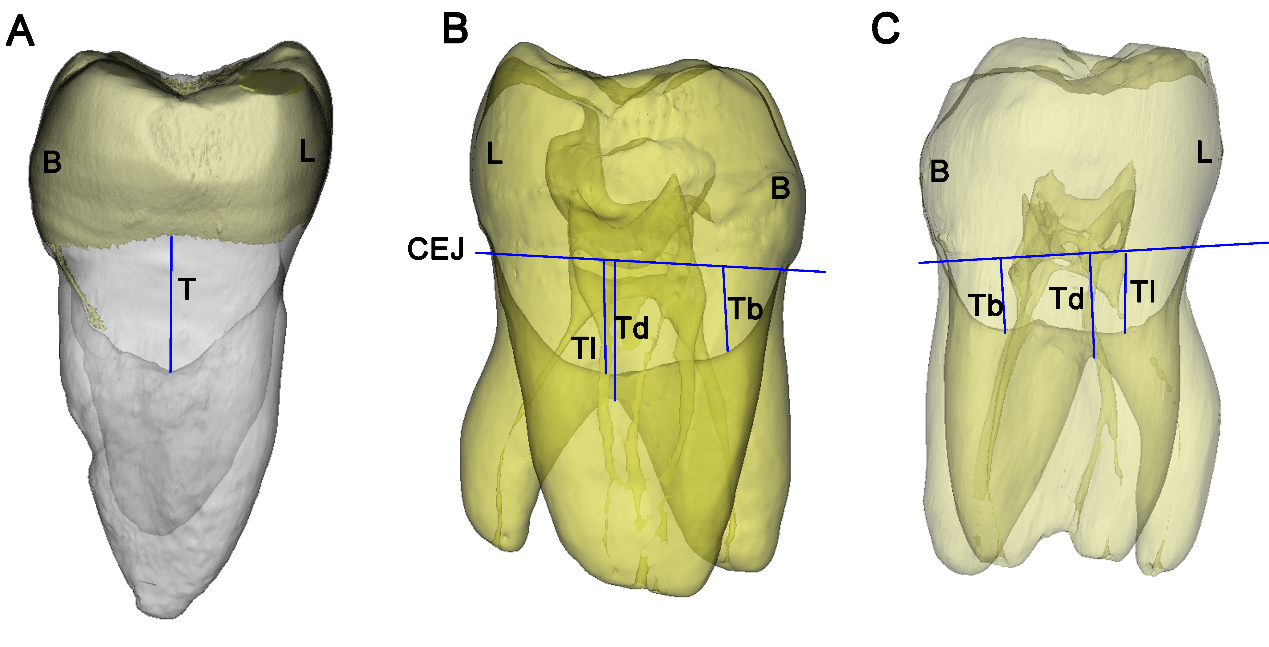


**Fig. 2** Classification of root furcation and measurement of root trunk length. **a** A two-rooted mandibular first molar with a type V root furcation (L is the root trunk length). **b** A three-rooted mandibular first molar with a type U root furcation (Ll is bucco-lingual root trunk length and Ld is distal root trunk length). **c** A three-rooted mandibular first molar with a type W root furcation (Lb and Ll are buccal and lingual root trunk length, respectively, and the longer one [Ll] is regarded as the bucco-lingual root trunk length). CEJ is cement-enamel junction.


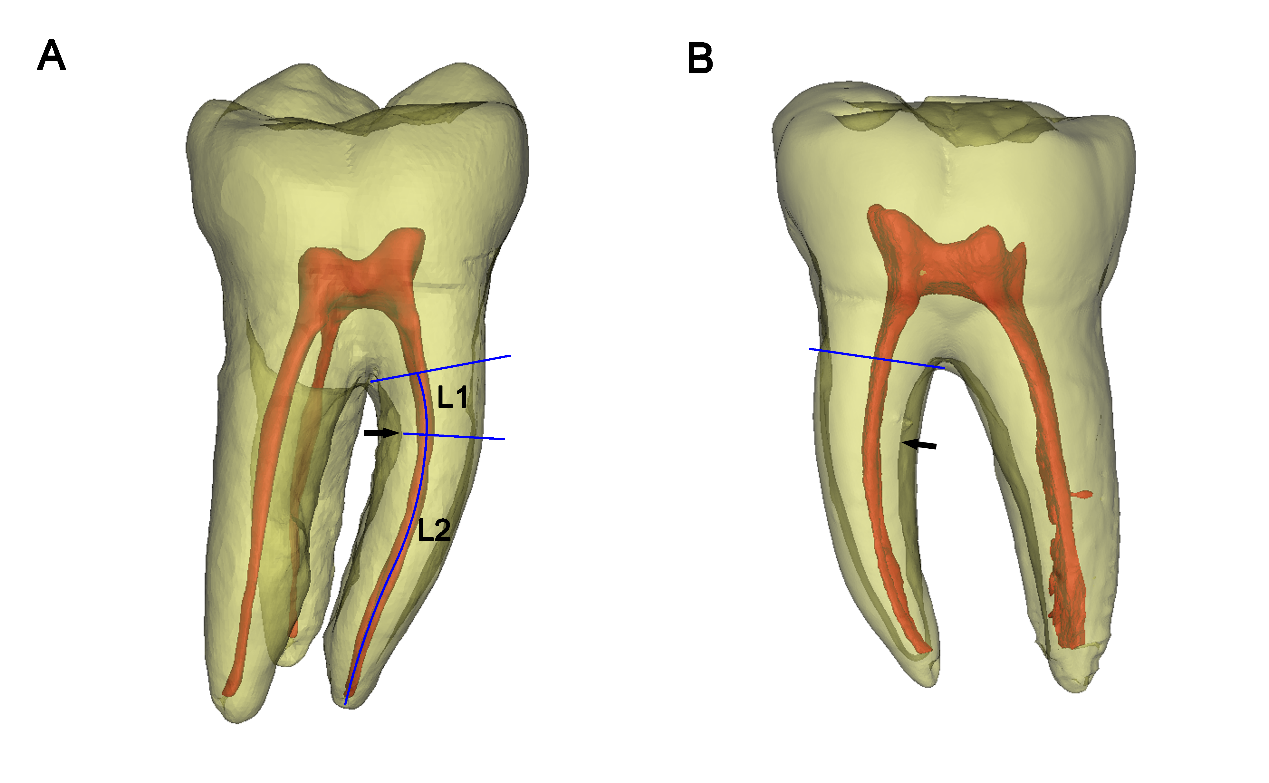


**Fig. 3** The maximum depth of the distal concavities of the mesial roots of mandibular first molars. **a** A root concavity is present at the distal side of mesial root of a three-rooted mandibular first molar (the arrow indicates the deepest site of the root concave, which corresponds to the locally thinnest canal wall thickness). **b** A double-rooted mandibular first molar (the arrow indicates the deepest site of the root concave, and the distal canal wall thickness decreases towards the apex).


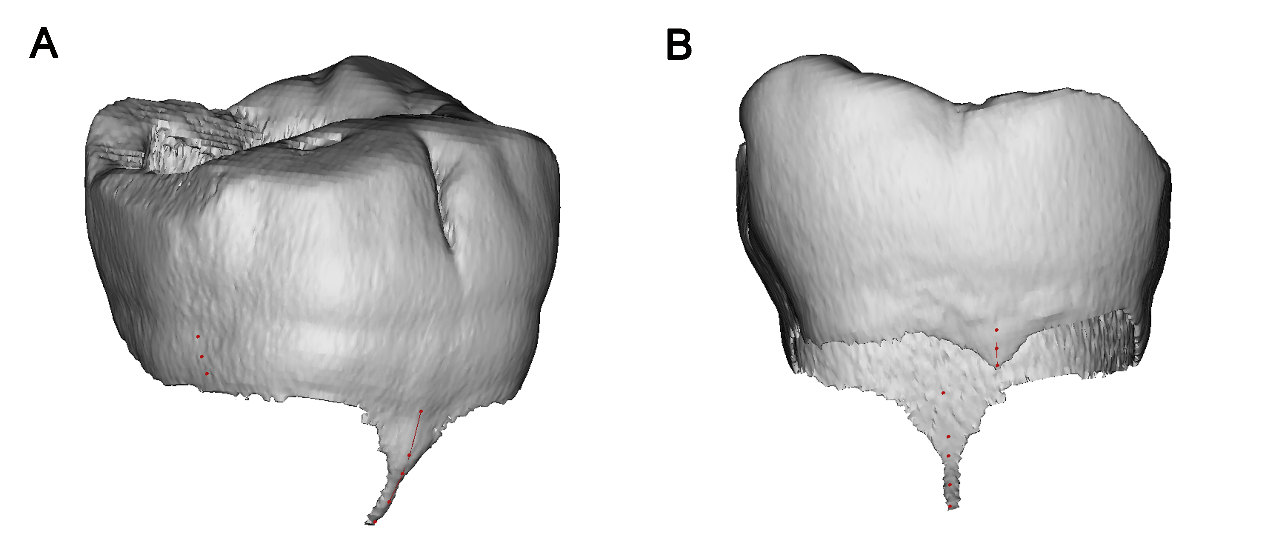


**Fig. 4** Measurement of buccal and lingual enamel projections. **a** Buccal view. **b** Lingual view.





**Fig. 5** Measurement results of furcation angles of two- and three-rooted mandibular first molars (error bar is SD; * is *p* < 0.05; ** is *p* < 0.01).





**Fig. 6** Measurement results of the length of buccal/lingual enamel projections in two- and three-rooted mandibular first molars (error bar is SD; ** is *p* < 0.01).

**Tables:**

**Table 1** The measurement result of the angle of root furcation

| **Tooth group** | **View** | **Furcation angle**  **between 2 roots** | ***n*** | **Angle (degrees)** | |
| --- | --- | --- | --- | --- | --- |
|  |  |  |  | **Mean±SD** | **Range** |
| 3RM1 | Buccal | M-DB | 25 | 34.7±10.7^b^ | 12.3-55.3 |
|  | Buccal | M-DL | 25 | 25.4±8.7^c^ | 9.0-47.2 |
|  | Distal | DB-DL | 25 | 59.2±12.8^a^ | 30.0-80.8 |
| 2RM1 | Buccal | M-D | 25 | 29.5±9.2^bc^ | 11.3-46.7 |

The values with the different lowercase superscript letters along the same column are significantly different (*p* < 0.05).

**Table 2** Classification of the root furcation *n*（%）

| **Tooth group** | ***n*** | **Type V** | **Type U** | **Type W** |
| --- | --- | --- | --- | --- |
| 3RM1 | 25 | 14（56.0） | 6（28.0） | 5（20.0） |
| 2RM1 | 25 | 13（42.0） | 7（28.0） | 5（20.0） |
| Total | 50 | 27（54.0） | 13（26.0） | 10（20.0） |

The Chi-square test demonstrates no statistically significant differences between the two- and three-rooted tooth groups (*p* > 0.05)

**Table 3** The measurement results of the root trunk length

| **Tooth group** | **Location** | ***n*** | **Root trunk length (mm)** | |  |
| --- | --- | --- | --- | --- | --- |
|  |  |  | **Mean±SD** | **Range** |  |
| 3RM1 | Bucco-lingual | 25 | 3.01±0.52^b^ | 1.97-3.86 |  |
|  | Distal | 25 | 3.69±0.74^a^ | 2.18-4.85 |  |
| 2RM1 | Bucco-lingual | 25 | 2.99±0.53^b^ | 2.16-4.21 |  |

The values with the different lowercase superscript letters along the same column are significantly different (*p* < 0.01).

**Table 4** The measurement results of the maximum depth and site of the distal root concavities of mesial roots of mandibular first molars

| **Tooth group** | *n* | **Maximum depth of distal root concavities (mm)** | |  | **The site of the maximum depth of distal root concavities (mm below furcation)** | |
| --- | --- | --- | --- | --- | --- | --- |
|  |  | **Mean±SD** | **Range** |  | **Mean±SD** | **Range** |
| 3RM1 | 25 | 0.61±0.21 | 0.34-1.18 |  | 3.00±1.55 | 1.18-7.35 |
| 2RM1 | 25 | 0.70±0.16 | 0.51-0.99 |  | 2.68±1.04 | 0.96-5.24 |
| Total | 50 | 0.66±0.19 | 0.34-1.18 |  | 2.84±1.30 | 0.96-7.35 |

No significant difference was detected between the two tooth groups (*p* > 0.05).

**Table 5** The measurement results of the length of enamel projections (mm)

| **Tooth group** |  | ***n*** | **Buccal** | |  | **Lingual** | |
| --- | --- | --- | --- | --- | --- | --- | --- |
|  |  |  | **Mean±SD** | **Range** |  | **Mean±SD** | **Range** |
| 3RM1 |  | 25 | 3.25±1.60 | 0.65-4.96 |  | 0.71±0.27** | 0.32-1.35 |
| 2RM1 |  | 25 | 2.85±1.57 | 0.71-6.47 |  | 0.63±0.28** | 0.26-1.29 |
| Total |  | 50 | 3.05±8.65 | 0.65-6.47 |  | 0.67±8.65** | 0.26-1.35 |

**, *p* < 0.01 as compared between buccal and lingual sides. No significant difference was detected as compared between 3RM1 and 2RM1 groups (*p* > 0.05).

**Table S1** Classification of the root furcation in both genders *n*（%）

| **Gender** | ***n*** | **Type V** | **Type U** | **Type W** |
| --- | --- | --- | --- | --- |
| Male | 24 | 11（45.8） | 7（29.2） | 6（25.0） |
| Female | 26 | 16（61.5） | 6（23.1） | 4（15.4） |
| Total | 50 | 27（54.0） | 13（26.0） | 10（20.0） |

The Chi-square test demonstrates no statistically significant differences between the two genders (*X*^2^ = 1.325, *p* = 0.516)

**Table S2** Classification of the root furcation in two age groups *n*（%）

| **Age groups** | ***n*** | **Type V** | **Type U** | **Type W** |
| --- | --- | --- | --- | --- |
| < 50 years | 25 | 14（56.0） | 5（20.0） | 6（24.0） |
| ≥ 50 years | 25 | 13（52.0） | 8（32.0） | 4（16.0） |
| Total | 50 | 27（54.0） | 13（26.0） | 10（20.0） |

The Chi-square test demonstrates no statistically significant differences between the two age groups (*X*^2^ = 1.129, *p* = 0.569)
